# Supplementary material for: CdTiO3-NPs incorporated TiO2 nanostructure photocatalyst for scavenger-free water splitting under visible radiation
Source: PLoS One. 2022 Oct 18;17(10):e0276097. doi: 10.1371/journal.pone.0276097 (PMC9578619; doi:10.1371/journal.pone.0276097)
Supplement: Attachment — Submitted filename: Response 01.docx [file pone.0276097.s001.docx]

**Dear Dr. Satya Pal Nehra**

**Academic Editor**

**PLOS ONE**

Thank you for your kind response about the manuscript [PONE-D-22-20788] titled:

**“**CdTiO_3_ NPs-incorporated TiO_2_ nanostructures as effective photocatalyst for possible scavenger-free water photo splitting process under visible light radiation**”**

The referee's comments were helpful to strength the manuscript. We would like to inform you that we have modified the manuscript according to the newly given comments.

To make it more easily, we have written the comments in bold phase followed by the responses in normal one. Moreover, in the revised manuscript, you can find the changes in the text in blue color.

We hope our responses cover all the comments. It will be our pleasure to respond about any more comments.

Thank you for your cooperation

Sincerely yours

Corresponding author

Nasser A. M. Barakat

Professor

Chemical engineering dep., Minia university, Egypt

**Reviewer #1:**

**Erfan and co-author created a very interesting and valuable study dealing with the preparation of Cd(II) dopped TiO_2_ catalysts in different ratios. The photocatalytic potentials of prepared materials were investigated in the hydrogen production by water splitting reaction. The presented manuscript can be considered for publication in PLOS ONE after major revision, addressing of following points:**

1- **Introduction: ... or the use of a photocatalyst for water splitting [4, 5]. ... List the following review dealing with hydrogen production using GCN: R. Sharma, M. Almáši, S.P. Nehra, V.S. Rao, A. Sharma, I.P. Jain, Photocatalytic hydrogen production using graphitic carbon nitride (GCN): A precise review, Renew. Sust. Energ. Rev. (2022) in press,** [**https://doi.org/10.1016/j.rser.2022.112776**](https://doi.org/10.1016/j.rser.2022.112776)

*Response:*
Thank you for this comment, adding this important review will enhance the manuscript.

The review has been added to the revised manuscript as reference 6

**2. Materials - It would be appropriate to unify information related to chemicals: (abbreviation, summary formula, Mw, purity). The chemical composition of cadmium acetate is not correct; 2 moles of water are missing.**

***Response:***

The reviewer is right; the chemicals information is unified in the updated manuscript version. Concerning cadmium acetate, it was out mistake, we have checked the used bottle and discovered that the reviewer is right; it associated with two water molecule so this mistake has been corrected in the revised manuscript.

**3-The volume of released hydrogen was not determined directly, e.g. GC, but indirectly by monitoring the change in water volume. During the experiments, a 1000 W lamp was used, which produced a lot of heat. How do the authors prevent water evaporation? Because the aforementioned evaporation of water introduces measurement error.**

***Response:*** As usual, the reviewer arises very valuable comment.

It is noteworthy mentioning that to ensure the collected gas represents the water splitting reaction product gases and no interfere from water vapor especially the used lamp becomes hot during illuminating time, these precautions have been conducted. First, the lamp was cooling by a suction fan placed above the lamp so this used fan sucks the hot air around the reactor flask. Second, the collecting gas beaker was placed a way from the lamp so it was always cold. Third, the used water in the collector beaker was checked continuously during the reaction time to be sure it still cold. Fourth, after every experiment, the collect gas was left for relatively long time to allow any water vapor to condensate; no any change in the gas volume was observed which indicates the collected gas is free from any water vapor.

*This paragraph was added in the revised manuscript.*  **4- The authors prepared three doped TiO_2_ materials with Cd(II), where cadmium acetate was added during the sol-gel synthesis in different ratios of 0.5; 1 and 2. But what is the real content of Cd(II) in the prepared solid samples? (possible solution: EDX, Rietveld analysis form PXRD, mineralization of materials in aqua regia and further AAS or ICP-MS measurements).
*Response:*** This comment came also from another mistake in the initial manuscript; we strongly appreciate the respected reviewer efforts.

To prepare final nanofiber mats containing 0.5, 1, and 2 wt.% CdO with respect to titanium element, the corresponding mass of cadmium acetate is added. The calculations were based on assumptions that a complete elimination of the used polymer and full decompositions of titanium isopropoxide and cadmium acetate di-hydrate precursors into titanium oxide and cadmium oxide, respectively will carry out. The calcination process was done in air atmosphere. Moreover, to ensure performing complete oxidation reactions, suction pump was used to remove the gaseous products from the reaction atmosphere as well as to fill the tubular furnace by fresh air continuously. Accordingly, the claiming about complete elimination of the used polymer and converting the used metallic precursors to the oxide forms is acceptable. Considering that the melting points of titanium oxide and cadmium oxide are 1843 and 900 ^o^C, respectively, no losses in the metal content are expected especially the calcination temperature was chosen to be 200 ^o^C less than the melting point of the relatively volatile oxide (CdO).

*This explanation has been added in the section ”2.2* ***Fabrication of TiO_2_ and Cd-doped TiO_2_ nanofibers and nanoparticles”*** *in the revised manuscript.*

*Moreover, EDX has been conducted (Fig. 3) in the revised manuscript with this explanation.*

“It is worth mentioning that EDX analysis has been conducted for 0.5 % sample. The numerical data summarized in the inset table supports the aforementioned calculation hypotheses. Therefore, it can be concluded that, during the calcination process, the used polymer has been completely eliminated and the metallic precursors have been changed into the oxide forms without considerable losses in the metals content**”**

**5- From an environmental point of view, the authors should investigate the possible leaching of Cd(II) toxic ions into the water after water splitting reaction (e.g. ICP-MS or AAS). Moreover, the H2 evolution rate of Cd(II) doped samples decreases with time compared to pure TiO2, which also indicates possible leaching.**

***Response:*** It is also good comment. Unfortunately, due to COVID-19 pandemic we could not do some analyses. However, the stability of proposed photocatalysts was investigated through checking the stability at different successive cycles. In the revised manuscript, Figure 6 was newly added. In this figure, 0.5 Cd nanofibers were used for three successive cycles. As shown in the figure, almost no change in the activity can be observed which denotes high stability of the proposed photocatalyst.

**6- What is the effectiveness (recyclability) of the catalyst (e.g. material with 0.5 Cd(II) content) after multiple uses (for example, 5 cycles)?**

***Response:***

To provide a proper response for the given comment, Fig. 6 has been updated to investigate the stability of 0.5% Cd nanofiber. The discussion has been updated as follow:

Reusability is an important feature for the photocatalysts. Figure 6 demonstrates the obtained results after using the 0.5 % Cd nanofiber for three successive cycles. As figure 6 manifested, the nanofiber exhibited sustainable photocatalytic performance even after three cycles without significant loss of H_2_ generation rate. These results confirm that the electrospun product was stable and reusable. Furthermore, such nanofibers could be effortlessly separated from the solution by facile sedimentation as the mixing stopped benefiting from the instinct feature of the large aspect ratio.

**Reviewer #2:**

**The selected topic covers a sufficient range of general interest including at the same time novel scientific aspects. The experimental setup is well presented along with the interpretation of the obtained data. Diagrams and images are adequately attached and the literature included is spherical enough of what's been done so far. Further a few suggestions are from my side to make the manuscript complete:**

**We strongly appreciate the great efforts of the respected reviewer in evaluating the manuscript; the given comments were valuable to strength the manuscript.**

1- **The title seems too long, it’s good to limit it in 10-13 words. An example of suggested title from my side is “CdTiO3-NPs incorporated TiO2 nanostructure photocatalyst for scavenger free water splitting under visible radiation”. If you feel it retain the sense of your article, consider it.**

***Response:***

The suggested title is concise and meaningful so we changed the title in the revised manuscript accordingly.

2- **On Page 2., instead of writing “It is hypothesized that the formation of type I heterostructures between the TiO2 matrix and CdTiO3 nanoparticles….” Use “may be” to show your hypothesis.**

***Response:***

The text has been updated in the revised manuscript

3- **In Introduction section on Page 3, non-metallic doping lacked some new research. Some related latest literature should be updated, such as RSER,2022,168:112776(https://doi.org/10.1016/j.rser.2022.112776); Chemosphere,2022,305:135467(https://doi.org/10.1016/j.chemosphere.2022.135467); IJHE,2020,45:23937 (**[**https://doi.org/10.1016/j.ijhydene.2019.06.061**](https://doi.org/10.1016/j.ijhydene.2019.06.061)**).**

***Response:***

It is believed that the suggested references strength the manuscript, so they were cited in the revised version

4- **Do not use e/h, rather put symbols also (e-/h).**

***Response:***

The text has been updated in the revised version
